# Phylogenetic analysis of *Uncaria* species based on internal transcribed spacer (ITS) region and ITS2 secondary structure

**DOI:** 10.1080/13880209.2018.1499780

**Published:** 2018-11-04

**Authors:** Shuang Zhu, Qiwei Li, Shanchong Chen, Yesheng Wang, Lin Zhou, Changqing Zeng, Jun Dong

**Affiliations:** a Center for Bioresources and Drug Discovery and School of Biosciences and Biopharmaceutics Guangdong Province Key Laboratory for Biotechnology Drug Candidates, Guangdong Pharmaceutical University, Guangzhou, People’s Republic of China;; b School of Traditional Chinese Medicine, Guangdong Pharmaceutical University, Guangzhou, People’s Republic of China

**Keywords:** Molecular phylogeny, discrimination, DNA barcoding

## Abstract

**Context:** The plant genus *Uncaria* (Rubiaceae), also known as Gouteng, is the source of an important traditional Chinese medicine. Misidentification and adulteration of Gouteng affect the safety and efficacy of the medication. Phylogenetic relationships among the species of this genus are unknown.

**Objective:** The present study sought to detect the phylogenetic relationships based on internal transcribed spacer (ITS) region of all 12 species of *Uncaria* recorded in the *Flora of China*.

**Materials and methods:** Accession of seven species of *Uncaria* served as reference samples. ITS region was used for polymerase chain reaction (PCR) amplification of the reference samples representing 39 specimens. Distance analysis, species discrimination, and secondary structure of ITS2 were used to assess the ability of ITS sequence in authenticating. The phylogenetic relationships were detected using three methods: Bayesian inference (BI), maximum likelihood (ML), and neighbor joining (NJ).

**Results:** Five species of traditional Chinese medicine Gouteng were well resolved in molecular phylogenetic tree. Besides, *Uncaria lancifolia* Hutch. was closer to *U. rhynchophylloides* F.C. How and *U.* sessilifructus Roxb. was closer to U. laevigata Wall. within the tree. Further, we also found that ITS2 secondary structure can be a candidate tool in distinguishing two closely related species *U. yunnanensis* K.C.Hsia and *U. lanosa* Wall. For accurate identification of different species of *Uncaria* based on species-specific nucleotide sites, a consensus sequences database with all 12 species is established.

**Discussions and conclusions:** The results are able to discriminate *Uncaria* species and illustrate the phylogenetic relationships, which are essential for the investigation of adulterants and misidentifications of *Uncaria*.

## Introduction

*Uncaria* (Rubiaceae) is a genus that contains 34 species: 29 in tropical Asia through Australia, three in Africa and Madagascar, two in tropical America and 12 species (five endemics) in China (Ridsdale 1978; Tao et al. 2011). The Chinese pharmacopeia records the stems with hooks from five *Uncaria* species namely, *Uncaria rhynchophylla* Miq., *U, macrophylla* Wall., *U. hirsute* Havil., *U. sinensis* Havil, and *U. sessilifructus* Roxb., which form an important part of traditional Chinese medicine known collectively as Gouteng (Chinese Pharmacopoeia Commission 2015). Previous studies have reported that the main compounds in Gouteng species (alkaloids, triterpenes, and uncarinic acids) have several beneficial properties, such as being antihypertensive, analgesic, sedative, and antioxidant (Ridsdale 1978; Song et al. 2000; Kang et al. 2015; Pan et al. 2015). The potential pharmacological activity from *Uncaria* continues to develop, particularly in the area of immunomodulatory, anti-inflammatory, and antitumor (Lee et al. 2000; Yuan et al. 2009; Lima-Junior et al. 2013; Zhang et al. 2013). Moreover, Gouteng is a traditional herb used in northeast Asia for the treatment of Parkinson’s disease (Shim et al. 2009; Chen 2016). The chemical composition and bioactivities vary among species: *U. rhynchophylla* and *U. sinensis* have the highest quality of beneficial compounds and are the most important medicine resources of Gouteng in China (Kang et al. 2015). However, due to their extensive collection for medicinal use, plants of high value species are now endangered in some regions of China. Moreover, the medicinal herb Gouteng is being misidentified and adulterated with similar but less valuable species such as *Uncaria laevigata* Wall., *U. lancifolia* Hutch., and *Uncaria scandens* Hutch., which is affecting the safety and efficacy of the medication.

The identification of *Uncaria* (Gouteng) is based primarily on the morphological characteristics, microscopic structures and/or chemical components of the plant (Gao et al. 2015). Previous studies have used restriction fragment length polymorphism (RFLP) and random amplified polymorphic DNA (RAPD) to look for molecular markers in *Uncaria* (Xu et al. 2012; Zhu, Zhou et al. 2012). Zhao et al. (2018) demonstrated that the liquid chromatography–mass spectrometry tandem ion trap-time of flight mass spectrometry (LCMS-IT-TOF) was a feasible method to solve the confusion in *Uncaria* species. Tang et al. (2016) evaluated five different DNA barcodes and proposed ITS2 and *psb*A*–trn*H as suitable markers for identification in this genus, but the phylogenetic relationships of *Uncaria* are still not fully known, especially among the five medicinal species recorded in the Chinese pharmacopeia.

DNA barcoding using short genetic markers or gene regions for species identification has potential for use in the detection and protection of endangered and valuable species (Hebert et al. 2003; CBOL Plant Working Group 2009; Hollingsworth et al. 2011). The Consortium for the Barcode of Life (CBOL) has proposed combined plastid barcoding with *mat*K and *rbc*L genes as an alternative for species identification among plants (CBOL Plant Working Group 2009). Several chloroplast gene sequences such as *psb*A*–trn*H, *trn*L*–trn*F, *ycf1*, and *rpoC1* have been evaluated as potential DNA barcodes (CBOL Plant Working Group 2009; Dong et al. 2015; Yu et al. 2016). In addition to plastid barcoding, internal transcribed spacer (ITS) regions of ribosomal genes have been proposed as supplemental barcodes for *mat*K and *rbc*L. The ITS region includes the ITS1 and ITS2 regions, separated by the 5.8S gene, and is situated between the 18S and 28S genes in the nrDNA repeat unit (Bellemain et al. 2010). Despite problems associated with the ITS region such as incomplete concerted evolution, fungal contamination, and difficulties in amplifying and sequencing in some species, it provides enough variable sites for differentiating among species (Chen et al. 2010; Yao et al. 2010; China Plant BOL Group 2011; Lee et al. 2016; Wang et al. 2017). Compared to the ITS region, the ITS2 region is easy to amplify and sequence, and also provides sufficient information for phylogenetic analysis. Moreover, the secondary structure of the ITS2 region can offer additional information for species identification. The ITS2 RNA transcript contains a core structure of two helices with hallmark characteristics that are important for ribosomal RNA processing (Coleman 2007). This secondary structure allows the detection of sequencing errors and pseudogenes in the ITS2 region (Coleman 2007; Rampersad 2014).

In the present study, we tested the ability of ITS and ITS2 regions to discriminate among *Uncaria* species, and established a sequence database with species-specific positions to discriminate the 12 species of *Uncaria* recorded in the *Flora of China*. We also attempted to illustrate the phylogenetic relationships among 12 species of *Uncaria* and use ITS2 secondary structure to distinguish potential closely related species.

## Materials and methods

### DNA extraction, PCR amplification and sequencing

This study was based on a total of 39 *Uncaria* specimens comprised of 32 fresh or silica gel-dried leaf specimens and 7 stem specimens, which included 7 of 12 *Uncaria* species (Table 1). They were collected from various locations in China and identified by Professor Changqing Zeng (School of Traditional Chinese Medicine, Guangdong Pharmaceutical University) based on morphological traits.

**Table 1.** Detailed information about newly obtained ITS and ITS2 sequences from *Uncaria* specimens in the present study.

					ITS			ITS2		
Voucher	Tissue	Collection site	Putative identification	Molecular identification	GenBank accession	Length (bp)	GC (%)	GenBank accession	Length (bp)	GC (%)
GT-1	Leaf	Dinghu Mountain, Zhaoqing, Guangdong Province, China	*U. rhynchophylloides*	*U. rhynchophylloides*	MF033267	677	61.6	MF033306	220	66.4
GT-16	Stems	Dinghu Mountain, Zhaoqing, Guangdong Province, China	*U. rhynchophylloides*	*U. rhynchophylloides*	MF033268	677	61.7	MF033307	220	66.4
GT-35	Leaf	Dinghu Mountain, Zhaoqing, Guangdong Province, China	*U. rhynchophylloides*	*U. rhynchophylloides*	MF033269	677	61.6	MF033308	220	66.4
GT-8	Stems	Guangzhou University of Chinese Medicine, Guangdong Province, China	*U. rhynchophylla*	*U. rhynchophylla*	MF033270	677	61	MF033309	220	65
GT-17	Leaf	Yangshan, Qingyuan, Guangdong Province, China	*U. rhynchophylla*	*U. rhynchophylla*	MF033271	677	61	MF033310	220	65
GT-18	Leaf	Wengyuan, Shaoguan, Guangdong Province, China	*U. rhynchophylla*	*U. rhynchophylla*	MF033272	677	61	MF033311	220	65
GT-19	Leaf	Guiyang University of Chinese Medicine, Guizhou Province, China	*U. rhynchophylla*	*U. rhynchophylla*	MF033273	677	61	MF033312	220	65
GT-20	Leaf	Meizhou, Guangdong Province, China	*U. rhynchophylla*	*U. rhynchophylla*	MF033274	677	61	MF033313	220	65
GT-21	Leaf	Yangshan, Qingyuan, Guangdong Province, China	*U. rhynchophylla*	*U. rhynchophylla*	MF033275	677	60.9	MF033314	220	65
GT-22	Leaf	Wuzhou, Guangxi Province, China	*U. rhynchophylla*	*U. rhynchophylla*	MF033276	677	61	MF033315	220	65
GT-24	Leaf	Guangxi botanical garden, Guangxi Province, China	*U. rhynchophylla*	*U. rhynchophylla*	MF033277	677	61	MF033316	220	65
GT-25	Leaf	Guangzhou University of Chinese Medicine, Guangdong Province, China	*U. rhynchophylla*	*U. rhynchophylla*	MF033278	677	61	MF033317	220	65
GT-26	Leaf	Dapu, Meizhou, Guangdong Province, China	*U. rhynchophylla*	*U. rhynchophylla*	MF033279	677	61	MF033318	220	65
GT-27	Leaf	Tianzhu, Guizhou Province, China	*U. rhynchophylla*	*U. rhynchophylla*	MF033280	677	61	MF033319	220	65
GT-28	Leaf	Majiang, Guizhou Province, China	*U. rhynchophylla*	*U. rhynchophylla*	MF033281	677	61.2	MF033320	220	65
GT-31	Leaf	Kaiyang, Guizhou Province, China	*U. rhynchophylla*	*U. rhynchophylla*	MF033282	677	60.9	MF033321	220	65
GT-32	Leaf	Guangxi botanical garden, Guangxi Province, China	*U. rhynchophylla*	*U. rhynchophylla*	MF033283	677	60.9	MF033322	220	64.5
GT-H	Leaf	Jinfoshan, Chongqing, China	*U. rhynchophylla*	*U. rhynchophylla*	MF033284	677	61.3	MF033323	220	65
GT-Hunan	Leaf	Xinhua, Hunan Province, China	*U. rhynchophylla*	*U. rhynchophylla*	MF033285	677	61	MF033324	220	65
GT-GZY	Leaf	Guangzhou University of Chinese Medicine, Guangdong Province, China	*U. rhynchophylla*	*U. rhynchophylla*	MF033286	677	60.9	MF033325	220	64.5
GT-MZh	Leaf	Meizhou, Guangdong Province, China	*U. rhynchophylla*	*U. rhynchophylla*	MF033287	677	61	MF033326	220	65
GT-4	Leaf	Jinfoshan, Chongqing, China	*U. sinensis*	*U. sinensis*	MF033288	677	60.9	MF033327	220	64.1
GT-6	Stems	Guangxi botanical garden, Guangxi Province, China	*U. sinensis*	*U. sinensis*	MF033289	677	60.9	MF033328	220	64.1
GT-7	Stems	Guizhou Province, China	*U. sinensis*	*U. sinensis*	MF033290	677	60.9	MF033329	220	64.1
GT-34	Leaf	Anshun, Guizhou Province, China	*U. sinensis*	*U. sinensis*	MF033291	677	61	MF033330	220	64.5
GT-M	Leaf	South China botanical garden, Guangdong Province, China	*U. hirsute*	*U. homomalla*	MF033292	677	61.4	MF033331	220	65
GT-2	Leaf	South China botanical garden, Guangdong Province, China	*U. rhynchophylla*	*U. hirsuta*	MF033293	676	61.4	MF033332	220	66.4
GT-12	Leaf	Guangxi botanical garden, Guangxi Province, China	*U. hirsute*	*U. hirsuta*	MF033294	676	61.4	MF033333	220	66.4
GT-13	Stems	Dinghu Mountain, Zhaoqing, Guangdong Province, China	*U. hirsute*	*U. hirsuta*	MF033295	676	61.4	MF033334	220	66.4
GT-10	Leaf	Guangxi botanical garden, Guangxi Province, China	*U. sessilifructus*	*U. sessilifructus*	MF033296	678	61.9	MF033335	221	66.1
GT-15	Stems	Guangxi botanical garden, Guangxi Province, China	*U. sessilifructus*	*U. sessilifructus*	MF033297	678	61.9	MF033336	221	66.1
GT-23	Leaf	South China botanical garden, Guangdong Province, China	*U. sessilifructus*	*U. sessilifructus*	MF033298	678	61.9	MF033337	221	66.1
GT-30	Leaf	Guangxi botanical garden, Guangxi Province, China	*U. sessilifructus*	*U. sessilifructus*	MF033299	678	61.9	MF033338	221	66.1
GT-B	Leaf	South China botanical garden, Guangdong Province, China	*U. sessilifructus*	*U. sessilifructus*	MF033300	676	61.8	MF033339	221	66.1
GT-3	Leaf	Dinghu Mountain, Zhaoqing, Guangdong Province, China	*U. rhynchophylla*	*U. macrophylla*	MF033301	676	62.9	MF033340	220	67.3
GT-11	Leaf	Guangxi botanical garden, Guangxi Province, China	*U. macrophylla*	*U. macrophylla*	MF033302	676	62.7	MF033341	220	67.3
GT-14	Stems	Dinghu Mountain, Zhaoqing, Guangdong Province, China	*U. macrophylla*	*U. macrophylla*	MF033303	676	62.6	MF033342	220	67.3
GT-29	Leaf	Guangxi botanical garden, Guangxi Province, China	*U. macrophylla*	*U. macrophylla*	MF033304	676	62.7	MF033343	220	67.3
GT-33	Leaf	Wuming, Nanning, Guangxi Province, China	*U. hirsute*	*U. macrophylla*	MF033305	676	62.9	MF033344	220	67.3

Total DNA was extracted from fresh or silica gel-dried leaf/stem tissue using the Plant Genomic DNA Kit (Bioteke Co., Guangzhou, China) according to the manufacturer’s instructions. A primer pair (forward-ITS5: 5′-GGAAGTAAAAGTCGTAACAAGG-3′, reversed-ITS4: 5′-TCCTCCGCTTATTGATATGC-3′) was used for PCR amplification on an S1000 Thermal Cycler (BIO-RAD, USA). The PCR cycle consisted of 3 min at 95 °C, 30 cycles of 1 min at 94 °C, 50 s at 56 °C, 55 s at 72 °C, and 10 min at 72 °C. PCR products were purified and bidirectional sequenced using the same primer pair in Beijing Genomic Institute (Guangzhou, China). The ITS sequences obtained by sequencing were edited and refined manually using the software DNAStar version 7.1.

### Sequence analysis, distance analysis and species discrimination

The ITS sequences of the genus *Uncaria* were queried and downloaded from the National Center for Biotechnology Information (NCBI) database. Low quantity and ambiguous sequences were manually checked and deleted. Further, a total of 39 ITS sequences were obtained in this study. Each sequence was individually defined for a complete ITS2 fragment using the “Annotate” feature of the ITS2 database website (http://its2.bioapps.biozentrum.uni-wuerzburg.de/) (Schultz et al. 2006; Keller et al. 2009). DNA sequences generated from this study along with those downloaded from GenBank were assembled and aligned using the software MEGA version 6.06 (Tamura et al. 2013). The ITS dataset was used for nucleotide composition analysis, distance analysis, species discrimination and phylogenetic analysis, while the ITS2 dataset was used for secondary structure analysis. The nucleotide composition in the ITS region of each species was generated using MEGA version 6.06. Intraspecific and interspecific distances were calculated with the Kimura 2-Parameter (K2P) method using the software TaxonDNA version 1.8 (Kimura 1980; Meier et al. 2006). Barcoding gaps (i.e., the distribution of the pairwise intra- and interspecific distances) were illustrated by bar graphs. Species discrimination was calculated using the “Best Match” and “Best Close Match” functions in TaxonDNA, based on the K2P method and a minimum sequence overlap of 100 bp.

### Phylogenetic analysis

The sequence datasets were analyzed with three phylogenetic methods: Bayesian inference (BI), maximum likelihood (ML) and neighbor joining (NJ). Bayesian inference was performed with the computer program MrBayes ver. 3.2.6 (Ronquist et al. 2012) under the general time reversible (GTR)+G nucleotide substitution models determined by the program Mr Model test ver. 2.3 (Nylander 2004). Four simultaneous chains of Markov Chain Monte Carlo (MCMC) algorithm were run twice with two million generations. Trees were sampled every hundredth generation, whereby the first 5,000 trees were discarded as burn-in. The remaining trees were used to calculate posterior probabilities (PP) of the branching pattern in the 50% majority-rule consensus tree. The ML analyses, including 1,000 nonparametric bootstrap replicates, were carried out in MEGA version 6.06 under the Tamura 3-parameter (T92)+G model. The NJ tree was constructed under the K2P model, which is based on distance substitution, using MEGA version 6.06. Bootstrap support for the NJ tree was estimated with 1000 replicates, while uninformative characters (gaps and missing data) were completely deleted. Moreover, two *Nauclea* species were downloaded from GenBank database for use as outgroup species, as this genus is a relative of *Uncaria* (Manns and Bremer 2016).

### Prediction of secondary structure

The secondary structure of ITS2 was folded using the Mfold web server using the default temperature (37 °C) and ionic conditions (http://unafold.rna.albany.edu) (Zuker 2003). All output was used to predict a consensus secondary structure on the RNAalifold webserver (http://rna.tbi.univie.ac.at/cgi-bin/RNAWebSuite/RNAalifold.cgi), set at default options (Lorenz et al. 2011). Consensus ITS secondary structures were re-drawn as radial view structures and annotated using RNAlogo (http://rnalogo.mbc.nctu.edu.tw/index.php) (Chang et al. 2008). The compensatory base changes (CBCs) were detected using the program 4SALE version 1.7 (http://4sale.bioapps.biozentrum.uni-wuerzburg.de/) (Seibel et al. 2006, 2008).

## Results and discussion

### Sequences

The dataset used in this study included all 12 species of *Uncaria* recorded in *Flora of China*. The primer pair ITS5 and ITS4 effectively amplified the complete ITS sequences of all 39 *Uncaria* specimens, which were deposited in the NCBI GenBank database under the accession numbers MF033267 to MF033305. The ITS2 sequences (annotated and defined by the ITS2 database) were also submitted to GenBank (MF033306 to MF033344). The total length of newly obtained ITS and ITS2 regions ranged from 676 to 678 bp and from 220 to 221 bp, respectively. The GC content of the ITS region was slightly lower than that of the ITS2 region, i.e., 60.9–62.9% versus 64.1–67.3% (Table 1). Also, 93 *Uncaria* sequences of the ITS region were downloaded from the GenBank, and those sequences were also annotated and trimmed as ITS2 sequences. In total, two datasets (132 sequences of ITS and 132 sequences of ITS2) were obtained after combining the newly obtained sequences and downloaded sequences (Table 2; Supplemental Table S1). The ITS dataset contained 78 variable sites and 52 parsimony informative sites, while the ITS2 dataset comprised 39 variable sites and 27 parsimony informative sites.

**Table 2.** Summary statistics and species discrimination of ITS/ITS2 regions in *Uncaria*.

Items	ITS	ITS2
Individuals	132	132
Aligned length (bp)	679	222
Variable sites	78	39
Parsimony informative sites	52	27
Mean intraspecific distance (%)	0.23	0.32
Mean interspecific distance (%)	2.23	3.45
Mean GC content (%)	62	65.8
Best match		
Correct (%)	96.97	96.21
Ambiguous (%)	2.27	3.03
Incorrect (%)	0.75	0.75
Best close match		
Correct (%)	96.97	94.69
Ambiguous (%)	2.27	3.03
Incorrect (%)	0.75	0.75
Without any match closer than threshold (%)	0	1.51
Threshold (%)	0.82	0.92

Based on alignment of all *Uncaria* ITS sequences, the consensus sequences, species-specific nucleotide sites and degenerate nucleotides for each species are shown in Table 3. At least one species-specific site was found in each *Uncaria* species, while *U. yunnanensis* K.C. Hsia was highly similar to *U. lanosa* Wall. except for a single insertion of a thymidine at 602 bp in *U. yunnanensis*. Nucleotide comparison of consensus sequences is a reliable method that may be considered to identify closely-related, adulterated or misidentified *Uncaria* species, and be used to confirm morphological and chemical identification (Zhao et al. 2018). For example, when a query sequence was aligned to a consensus sequence of *Uncaria rhynchophylloides* F.C.How, a match of six sites (118 bp-C, 152 bp-G, 247 bp-A, 447 bp-T, 493 bp-T and 580 bp-C) identified the query sequence as *U. sinensis*.

**Table 3.** Species-specific nucleotide variation in the ITS region based on consensus sequences of different *Uncaria* species.

Species	Position																														
			1	1	1	1	1	2	2	2	4	4	4	4	4	4	4	4	4	5	5	5	5	5	5	5	6	6	6	6	6
	7	7	1	1	1	3	5	0	4	4	2	4	4	6	7	7	8	9	9	4	5	5	6	8	8	9	0	1	2	2	4
	5	9	6	7	8	6	2	9	2	7	7	5	7	5	8	9	3	2	3	6	0	8	5	0	6	9	2	1	0	2	1
*U. rhynchophylloides*	T	G	A	C	T	T	A	T	G	G	G	G	C	T	C	C	A	C	G	C	–	G	T	T	C	G	–	T	T	T	C
*U. rhynchophylla*	.	.	.	T	C	.	G	.	.	K	.	.	.	.	.	.	.	Y	.	.	–	.	.	C	.	.	–	.	.	.	.
*U. sinensis*	.	.	.	.	C	.	G	.	.	A	.	.	T	.	.	.	.	.	T	.	–	.	.	C	.	.	–	.	.	.	.
*U. homomalla*	.	.	.	.	C	.	G	.	.	.	.	.	.	.	T	.	.	.	.	.	–	.	.	C	.	T	–	.	.	.	.
*U. hirsuta*	.	T	.	.	C	.	G	.	A	.	.	.	.	C	.	.	.	.	.	.	–	.	.	C	.	.	–	.	.	.	.
*U. sessilifructus*	.	.	C	.	C	.	G	.	.	.	T	.	.	.	.	.	M	.	.	.	C	W	.	C	A	.	–	.	.	.	A
*U. macrophylla*	C	.	.	.	C	.	G	C	.	.	.	S	.	.	.	.	.	.	.	.	–	.	C	C	.	.	–	.	.	.	.
*U. laevigata*	.	.	R	.	C	.	G	.	.	.	.	.	.	.	.	.	G	.	.	A	–	.	.	C	.	.	–	.	.	.	.
*U. lancifolia*	.	.	.	.	Y	.	G	.	.	.	.	.	.	.	.	.	.	.	.	.	–	.	.	C	.	.	–	.	.	.	.
*U. lanosa*	.	.	.	.	C	C	G	.	.	.	.	.	.	.	.	.	.	.	.	.	–	.	.	C	.	.	–	T	C	A	.
*U. scandens*	.	.	.	.	C	.	G	.	.	.	.	.	.	.	.	T	.	.	.	.	–	.	.	C	.	K	–	.	.	.	.
*U. yunnanensis*	.	.	.	.	C	C	G	.	.	.	.	.	.	.	.	.	.	.	.	.	–	.	.	C	.	.	T	Y	C	A	.

Degenerate nucleotides: R: A/G, Y: C/T, M: A/C, K: G/T, S: G/C, W: A/T. Nucleotide numbering starts from 5′-TTTCCG.

### Distance analysis and species discrimination

**Figure 1.**
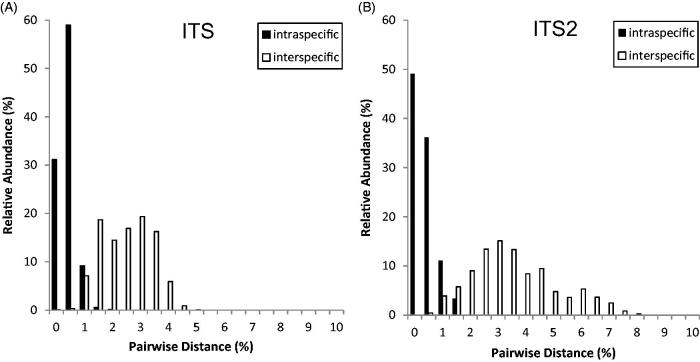
Distribution of intra- and interspecific distances for the ITS and ITS2 regions among dataset sequences.

The mean haplotype diversity and the mean nucleotide diversity of the ITS region of all *Uncaria* species were 0.954% and 1.967%, respectively. For the ITS2 region, the respective diversities were 0.932% and 2.932% (Table 4). *U. laevigata*, *U. lancifolia,* and *U. sessilifructus* had high mean nucleotide diversity in the ITS and ITS2 region. For a suitable barcode for species discrimination, the intraspecific distance is required to be lower than the interspecific distance, i.e., it should show a barcode gap (Meier 2008; Hartvig et al. 2015). The barcode gap analysis, based on uncorrected *p*-distance histograms, revealed a partial overlap between the intraspecific and interspecific distances of ITS and ITS2. The interspecific distances of sequences for ITS and ITS2 were considerably higher than the intraspecific distances (Figure 1). Moreover, the mean interspecific distances were significantly higher than the corresponding intraspecific distances for both ITS and ITS2 regions (Table 2).

**Table 4.** Intraspecific divergences of the ITS region in genus *Uncaria*.

	ITS				ITS2			
Species	No. of individuals	No. of haplotypes	Haplotype diversity (mean ± Std. dev.)	Nucleotide diversity (mean ± Std. dev. %)	No. of individuals	No. of haplotypes	Haplotype diversity (mean ± Std. dev.)	Nucleotide diversity (mean ± Std. dev. %)
*U. hirsuta*	11	7	0.541 ± 0.125	0.172 ± 0.046	11	4	0.463 ± 0.120	0.255 ± 0.074
*U. homomalla*	11	7	0.597 ± 0.118	0.183 ± 0.045	11	4	0.398 ± 0.122	0.285±±0.089
*U. laevigata*	6	5	0.667 ± 0.141	0.410 ± 0.104	6	5	0.667 ± 0.141	0.556 ± 0.155
*U. lancifolia*	8	4	0.775 ± 0.057	0.334 ± 0.055	8	3	0.625 ± 0.093	0.333 ± 0.067
*U. lanosa*	1	1	0	0	1	1	0	0
*U. macrophylla*	19	9	0.670 ± 0.076	0.167 ± 0.032	19	4	0.360 ± 0.089	0.188 ± 0.051
*U. rhynchophylla*	32	10	0.631 ± 0.066	0.203 ± 0.041	32	6	0.467 ± 0.072	0.277 ± 0.054
*U. rhynchophylloides*	3	2	0.533 ± 0.172	0.079 ± 0.025	3	1	0	0
*U. scandens*	11	6	0.476 ± 0.128	0.173 ± 0.068	11	5	0.407 ± 0.128	0.316 ± 0.124
*U. sessilifructus*	14	10	0.675 ± 0.098	0.321 ± 0.087	14	7	0.582 ± 0.105	0.606 ± 0.161
*U. sinensis*	11	5	0.749 ± 0.058	0.198 ± 0.024	11	3	0.255 ± 0.116	0.121 ± 0.057
*U. yunnanensis*	5	2	0.533 ± 0.095	0.088 ± 0.016	5	2	0.533 ± 0.095	0.242 ± 0.043
All *Uncaria* species	132	68	0.954 ± 0.006	1.967 ± 0.052	132	44	0.932 ± 0.007	2.932 ± 0.095

Species discrimination ability of the ITS and ITS2 region were tested by the best match and best close match methods (Table 2). These two methods are based on direct sequence comparison instead of tree-based identification. Best match assigns sequences with the smallest distance to the query sequence, while best close match requests best match sequences within 95% of the intraspecific distance (Meier et al. 2006). We found that we achieved a high rate of species discrimination in genus *Uncaria* using these two methods. The ITS region was able to correctly identify 128 out of 132 individuals (96.97%) using both best match and best close match. The correct identification rates of the ITS2 region were slightly lower than for the ITS region, yet still had a high discrimination ability (>94%). *U. lanosa* had only one sequence in the dataset, thus it could not be correctly identified due to the lack of conspecific matching.

Previous studies indicated that the ITS and ITS2 regions evolve rapidly, leading to genetic changes similar to those in plastid DNA barcodes (such as *mat*K, *rbc*L, *psb*A*-trn*H, et al.) (Kress et al. 2005; China Plant BOL Group 2011). However, the well-documented concerns and limitations of ITS include: (1) divergent paralogous copies within individuals lead by incomplete concerted evolution, (2) difficulties in amplifying and sequencing in some sample sets such as gymnosperms, and (3) fungal contamination (China Plant BOL Group 2011; Hollingsworth et al. 2011). Although they are imperfect, we believe that the ITS and ITS2 regions have sufficient ability to identify *Uncaria* species using best match and best close match techniques.

### Phylogenetic analysis within *Uncaria* species

**Figure 2.**
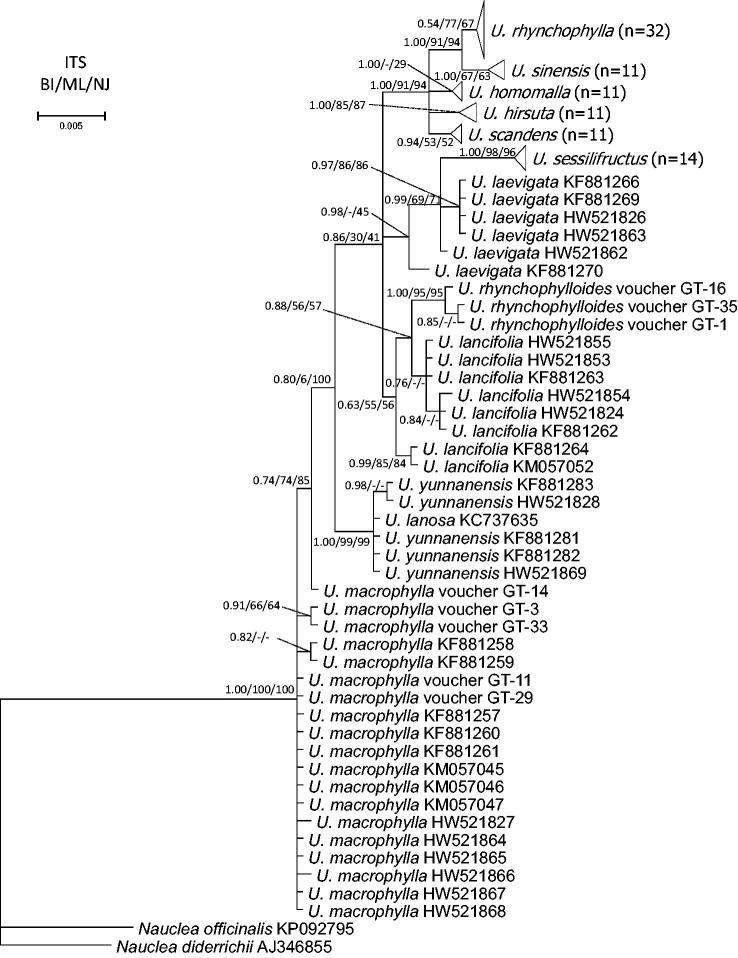
Phylogenetic tree inferred from the ITS region of *Uncaria*. Results from the maximum likelihood (ML) and neighbor-joining (NJ) bootstrap analyses were mapped onto the Bayesian inference (BI) tree. The node numbers indicated the Bayesian posterior probabilities and bootstrap value of ML and NJ. Some clades have been compressed to triangles due to more than ten conspecific individuals. The scale bar corresponds to 0.5 substitution per 100 nucleotide positions.

The ITS region is one of the most frequently utilized barcode in phylogenetic analysis at genus and species levels in eukaryotes (Coleman 2003; CBOL Plant Working Group 2009). We analyzed the ITS and ITS2 dataset with three different phylogenetic methods (BI, ML and NJ). The phylogenetic relationships that we identified among 12 species of *Uncaria* are shown in Figure 2 and Supplementary Figure S1. Phylogenetic trees inferred from the ITS and ITS2 dataset exhibited a structure similar to the branching patterns of the respective phylogenetic trees. The taxa *U. hirsute* Havil., *U. homomalla* Miq., and *U. scandens* were each clustered into separated clades, in both the ITS and ITS2 phylogenetic trees.

The phylogenetic relationships of five important medicinal species of *Uncaria* were shown in the present study (Figure 2). Three medicinal *Uncaria* plants, *U. rhynchophylla*, *U. sinensis* and *U. hirsuta*, are reportedly similar in the morphological characteristics of their hook-bearing stems, and therefore difficult to identify (Zhu, Chen, et al. 2012; Chinese Pharmacopoeia Commission 2015). In the present study, the taxa *U. rhynchophylla* and *U. sinensis* each clustered as monophyletic and as sister species in the ITS tree, while *U. sinensis* was nested with *U. rhynchophylla* in the ITS2 tree, and then clustered with three clades represented *U. hirsuta, Uncaria homomalla* and *U. scandens*. The other two medicinal plants were *U. macrophylla* and *U. sessilifructus. U. sessilifructus* was clustered as monophyletic and then clustered with *U. laevigata*. Except one individual (voucher GT-14), *U. macrophylla* was not clustered into a clade but separated from others species.

Ridsdale (1978) concluded that *U. rhynchophylloides* is the same species as *U. rhynchophylla* even though they appear morphologically distinct (Wang et al. 2007) and have different chemical constituents. *U. rhynchophylloides* has a high proportion of hirsutine but very low content of rhynchophylline and isorhynchophylline, which are important chemical compounds in medicinal Gouteng (Zhong and Feng 1996). In contrast, phylogenetic analysis in the present study showed that *U. rhynchophylloides* was not a synonym of *U. rhynchophylla,* as they were located far apart (Figure 2). On the other hand, *U. rhynchophylloides* was depicted as a separate clade and nested in *U. lancifolia,* which is highly similar to prior description of *U. rhynchophylloides* based on morphological characteristics (Wang et al. 2007), yet *U. lancifolia* did not cluster into a monophyletic clade in two trees. Similar branching patterns were also found between *U. sessilifructus* and *U. laevigata* (Figure 2), i.e., *U. sessilifructus* was clustered into a separate clade but *U. laevigata* failed to cluster into a monophyletic group. High nucleotide diversity may explain why *U. lancifolia* and *U. laevigata* were not monophyletic (Table 4).

Two highly similar species *U. yunnanensis* and *U. lanosa* clustered together into a monophyletic clade with high support rates in the ITS and ITS2 phylogenetic tree (Figure 2; Supplementary Figure S1). The only nucleotide insertion that occurred in *U. yunnanensis* (mentioned above) was deleted during construction of the phylogenetic tree.

To summarize, the ITS region has more variable sites to discriminate *Uncaria* species and thus has better discriminating performance than does the ITS2 region. The phylogenetic relationships of most *Uncaria* species were able to be resolved, except for *U. yunnanensis* and *U. lanosa*. In additional, the chloroplast DNA regions (*mat*K, *rbc*L, *psb*A-*trn*H, et al.) may be candidate barcodes to resolve the phylogenetic relationship between *U. yunnanensis* and *U. lanosa*.

### Secondary structure modeling and CBC analysis

**Figure 3.**
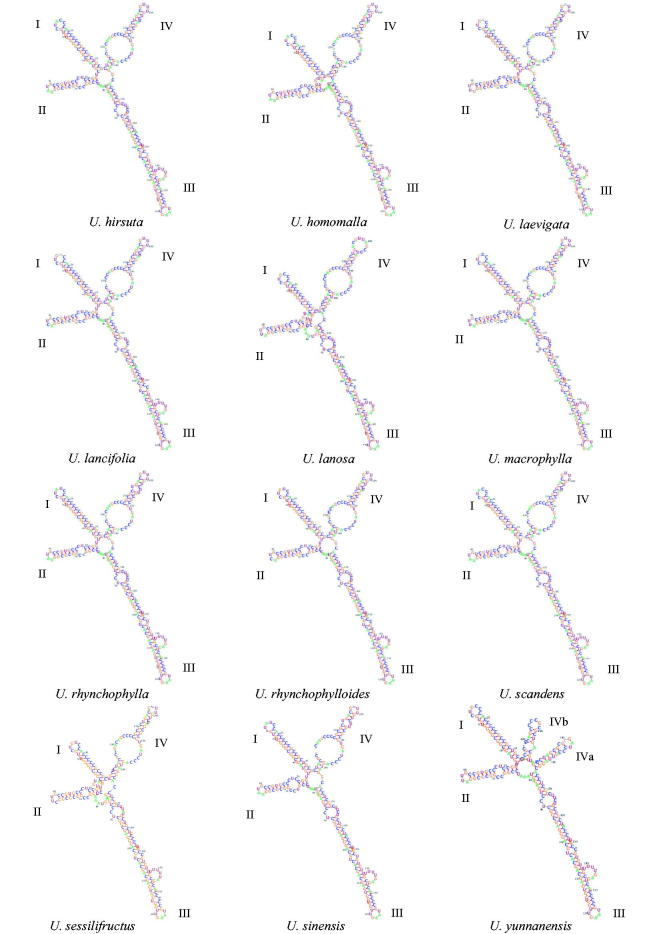
Putative secondary structures of ITS2 sequences in *Uncaria* species.

To predict ITS2 secondary structure, the minimum free energy method was used to form a structure synonymous with a natural-mode structure (Tinoco et al. 1971). The consensus secondary structure of most *Uncaria* taxa shared a similar folding pattern, i.e., four helices surrounding a central loop (Figure 3) which generated by the interaction of 5.8S-LSU (5.8S rRNA-28S rRNA) (Schultz et al. 2005; Coleman 2007). In order to discriminate the two closely related species *U. yunnanensis* and *U. lanosa*, we compared their secondary structure (Figure 3). The secondary structure of *U. lanosa* had a similar fold pattern as the consensus structure of others species, while *U. yunnanensis* showed a different fold pattern in helix IV (consisting of helix IVa and helix IVb). This difference may be caused by the species-specific insertion at 181 nt in the ITS2 molecule (at 602 nt in ITS region), which changed the fold pattern based on the minimum free energy method. Intraspecific mutation and prediction models can also induce the change of secondary structure. Hence, we also predicted the *U. yunnanensis* secondary structure using other programs or web servers [RNAstructure version 5.8.1; Reuter and Mathews 2010; RNAalifold webserver, and LocARNA webserver (http://rna.informatik.uni-freiburg.de/LocARNA/Input.jsp); Will et al. 2007; Smith et al. 2010; Will et al. 2012], and obtained similar results for the fold pattern (Supplementary Figure S2). Thus, we conclude that the secondary structure is a candidate method to discriminate *U. yunnanensis* and *U. lanosa*.

CBCs in the ITS2 molecule correlate with sexual incompatibility, so is an important molecular indicator for discriminating closely related species (Müller et al. 2007; Wolf et al. 2013; Shazib et al. 2016). However, it was reported that CBCs were not able to effectively discriminate among species (Caisová et al. 2013; Shazib et al. 2016). In the present study, we did not observe any CBCs in helices of the ITS2 molecule among *Uncaria* species, based on the 4SALE program (Supplementary Table S2). Thus, we conclude that CBC analysis is not an effective method to discriminate among *Uncaria* species.

## Conclusions

A comprehensive phylogenetic analysis including all the 12 species of *Uncaria* recorded in the *Flora of China* were concluded. Firstly, all *Uncaria* medicinal species could be clustered clearly in the molecular phylogeny tree. Secondly, we established the ITS database with all of obtained sequences and found that ITS sequences have appropriate variable sites for discrimination most of species in *Uncaria*. Finally, the ITS2 secondary structure can be used as candidate method for distinguishing the two closely related species *U. yunnanensis* and *U. lanosa*.

## Supplementary Material

Supplementary Materials
