# Supplementary material for: Phylogenetic analysis of Uncaria species based on internal transcribed spacer (ITS) region and ITS2 secondary structure
Source: Pharm Biol. 2018 Nov 4;56(1):548–58. doi: 10.1080/13880209.2018.1499780 (PMC6225500; doi:10.1080/13880209.2018.1499780)
Supplement: Supplementary Materials [file IPHB_A_1499780_SM1125.docx]

**Table S1.** GenBank accession numbers of downloaded sequences used in this study.

| Species | GenBank Accession |
| --- | --- |
| *U. rhynchophylloides* | - |
| *U. rhynchophylla* | HW521818, HW521829, HW521830, HW521831, HW521832, HW521833, HW521834, HW521835, HW521836, HW521837, HW521838, AJ346900, KF881265, KM057043 |
| *U. sinensis* | HW521819, HW521839, HW521840, HW521841, HW521842, FJ980386, KF881273 |
| *U. homomalla* | HW521822, HW521849, KF881250, KF881251, KF881252, KF881253, KF881254, KF881255, KF881256, KM057053 |
| *U. hirsute* | HW521823, HW521850, HW521851, HW521852, GU937110, KM057049, KM057050, KM057051 |
| *U. sessilifructus* | HW521825, HW521856, HW521857, HW521858, HW521859, HW521860, HW521861, KF881249, KM057048 |
| *U. macrophylla* | HW521827, HW521864, HW521865, HW521866, HW521867, HW521868, KF881257, KF881258, KF881259, KF881260, KF881261, KM057045, KM057046, KM057047 |
| *U. laevigata* | HW521826, HW521862, HW521863, KF881266, KF881269, KF881270 |
| *U. lancifolia* | HW521824, HW521853, HW521854, HW521855, KF881262, KF881263, KF881264, KM057052 |
| *U. lanosa* | KC737635 |
| *U. scandens* | HW521821, HW521845, HW521846, HW521847, HW521848, KF881274, KF881276, KF881277, KF881278, KF881279, KF881280 |
| *U. yunnanensis* | HW521828, HW521869, , KF881281, KF881282, KF881283 |
| *Nauclea diderrichii* | AJ346855 |
| *Nauclea officinalis* | KP092795 |

**Table S2.** CBC matrix detected by 4SALE program.

|  |  | 1 | 2 | 3 | 4 | 5 | 6 | 7 | 8 | 9 | 10 | 11 | 12 |
| --- | --- | --- | --- | --- | --- | --- | --- | --- | --- | --- | --- | --- | --- |
| 1 | *U. rhynchophylloides* |  | 0 | 0 | 0 | 0 | 0 | 0 | 0 | 0 | 0 | 0 | 0 |
| 2 | *U. rhynchophylla* | 0 |  | 0 | 0 | 0 | 0 | 0 | 0 | 0 | 0 | 0 | 0 |
| 3 | *U. sinensis* | 0 | 0 |  | 0 | 0 | 0 | 0 | 0 | 0 | 0 | 0 | 0 |
| 4 | *U. homomalla* | 0 | 0 | 0 |  | 0 | 0 | 0 | 0 | 0 | 0 | 0 | 0 |
| 5 | *U. hirsuta* | 0 | 0 | 0 | 0 |  | 0 | 0 | 0 | 0 | 0 | 0 | 0 |
| 6 | *U. sessilifructus* | 0 | 0 | 0 | 0 | 0 |  | 0 | 0 | 0 | 0 | 0 | 0 |
| 7 | *U. macrophylla* | 0 | 0 | 0 | 0 | 0 | 0 |  | 0 | 0 | 0 | 0 | 0 |
| 8 | *U. laevigata* | 0 | 0 | 0 | 0 | 0 | 0 | 0 |  | 0 | 0 | 0 | 0 |
| 9 | *U. lancifolia* | 0 | 0 | 0 | 0 | 0 | 0 | 0 | 0 |  | 0 | 0 | 0 |
| 10 | *U. lanosa* | 0 | 0 | 0 | 0 | 0 | 0 | 0 | 0 | 0 |  | 0 | 0 |
| 11 | *U. scandens* | 0 | 0 | 0 | 0 | 0 | 0 | 0 | 0 | 0 | 0 |  | 0 |
| 12 | *U. yunnanensis* | 0 | 0 | 0 | 0 | 0 | 0 | 0 | 0 | 0 | 0 | 0 |  |





**Figure S1.** Phylogenetic tree of the ITS2 region of the *Uncaria* species.


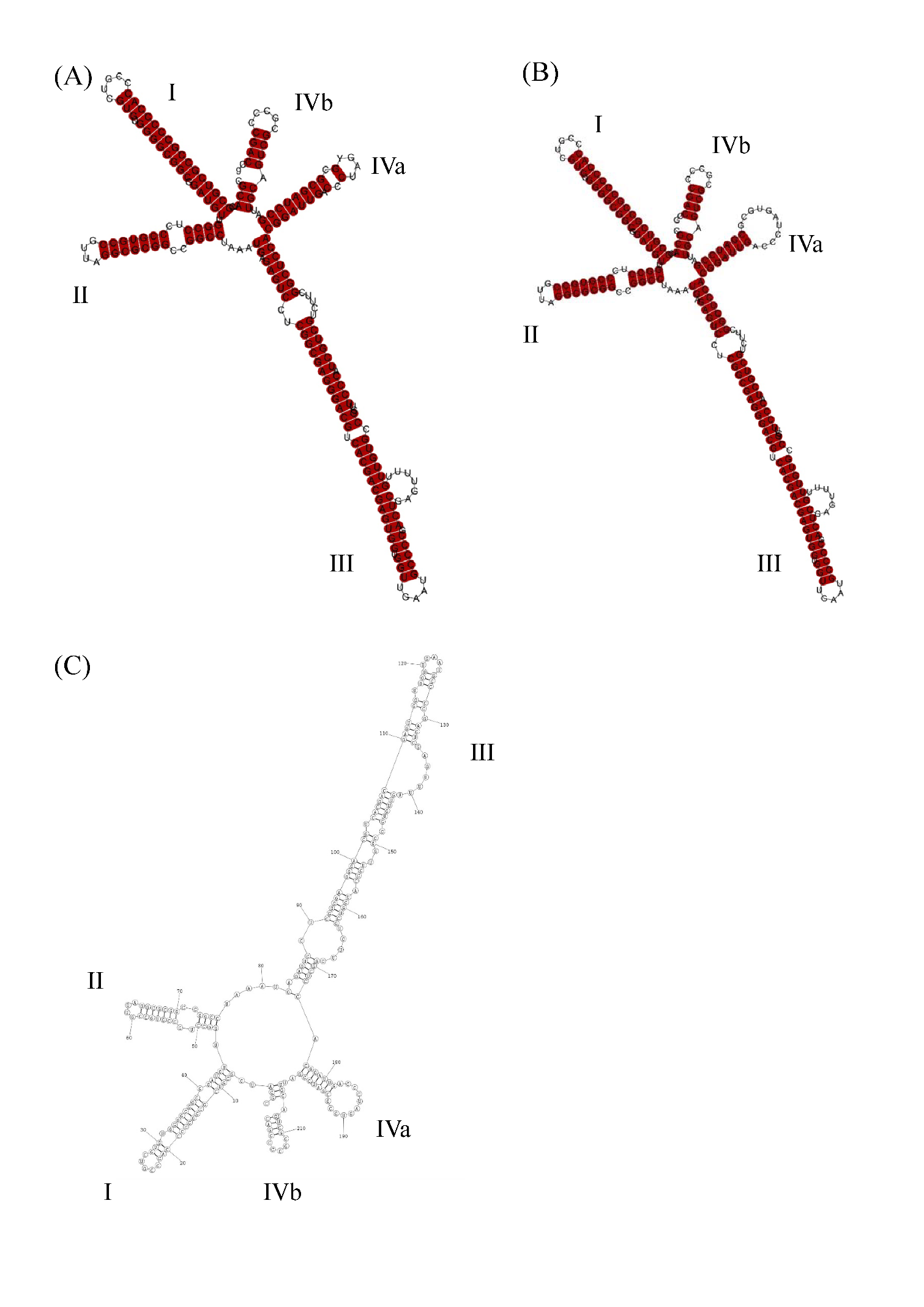


**Figure S2.** Consensus secondary structure of *U. yunnanensis*. The secondary structures were generated using (A) LocARNA, (B) RNAalifold and (C) RNAstructure, respectively.
